# Prevalence of prenatally diagnosed congenital cystic adenomatoid malformation among fetuses in China

**DOI:** 10.18632/oncotarget.18579

**Published:** 2017-06-20

**Authors:** Dazhi Fan, Qing Xia, Shuzhen Wu, Li Liu, Zhen Yu, Wen Wang, Song Wu, Xiaoling Guo, Zhengping Liu

**Affiliations:** ^1^ Foshan Institute of Fetal Medicine, Southern Medical University Affiliated Maternal & Child Health Hospital of Foshan, Foshan, Guangdong, 528000, China; ^2^ Department of Obstetrics, Southern Medical University Affiliated Maternal & Child Health Hospital of Foshan, Foshan, Guangdong, 528000, China; ^3^ Department of Epidemiology and Biostatistics, School of Public Health, Anhui Medical University, Hefei, Anhui, 230032, China; ^4^ Department of Library, The First Affiliated Hospital, College of Medicine, Zhejiang University, Hangzhou, Zhejiang, 310003, China; ^5^ Department of Maternal, Child and Adolescent Health, School of Public Health, Anhui Medical University, Hefei, Anhui, 230032, China; ^6^ School of Integrated Traditional and Western Medicine, Anhui University of Chinese Medicine, Hefei, Anhui, 230038, China

**Keywords:** congenital cystic adenomatoid malformation, fetuses, prevalence, meta-analysis

## Abstract

The prevalence of congenital cystic adenomatoid malformation among fetuses still varies in different studies in China. The present meta-analysis was intended to evaluate the pooled prevalence of fetuses in China. Four English (Pubmed, Elsevier Science Direct, Web of Science and the Cochrane Library) and four Chinese (the Chinese Biological Medical Literature database, the Chinese National Knowledge Infrastructure database, VIP database and Wanfang Data) databases were searched from inception to July 2016. Meta-analyses were performed using Stata (version 12.0), with prevalence and corresponding 95% confidence intervals using the random effect model. Five studies with 393496 fetuses were chosen for this meta-analysis. The overall pooled prevalence was 4.01/10000 (2.03/10000 - 6.00/10000) fetuses. Sensitivity analysis revealed that the results were stable, and Begg’s test and Egg’s test showed no potential risk of publication bias. This is, to our knowledge, the first study to systematically evaluate the literature of the prevalence of congenital cystic adenomatoid malformation among fetuses in China. Results showed that the prevalence among fetuses should be considerable. A large-scale multicenter study on the epidemiology across different areas in China is required.

## INTRODUCTION

Congenital cystic adenomatoid malformation (CCAM), also called congenital pulmonary adenomatoid malformation (CPAM), is the most common causative fetal lung lesions [1], firstly described by Ch’In and Tang in 1949 [2], and classified into three subtypes in 1977 [3], as well expanded into five types with a new name as CPAM by Stocker in 2002 [4]. In Canada, the condition occurs in one in 25000 to 35000 births [5]. Our previous study noted that the occurrence was approximately 3.34:10000 in China [6], nearly ten times higher than the level reported in Canada. Although previously studies provided lots of valuable information [7–9], epidemiological data on the prevalence of CCAM, especially in fetuses, is lacking and the prevalence of CCAM in fetuses also varies in different studies [10–12].

Therefore, we firstly present a pooled prevalence of CCAM for fetuses by conducting a systematic review of the literature published in China. Secondly, we also explore the prevalence according to different characters such as geographic distribution and maternal age in the subgroup.

## RESULTS

### Study characteristics

**Figure 1:**
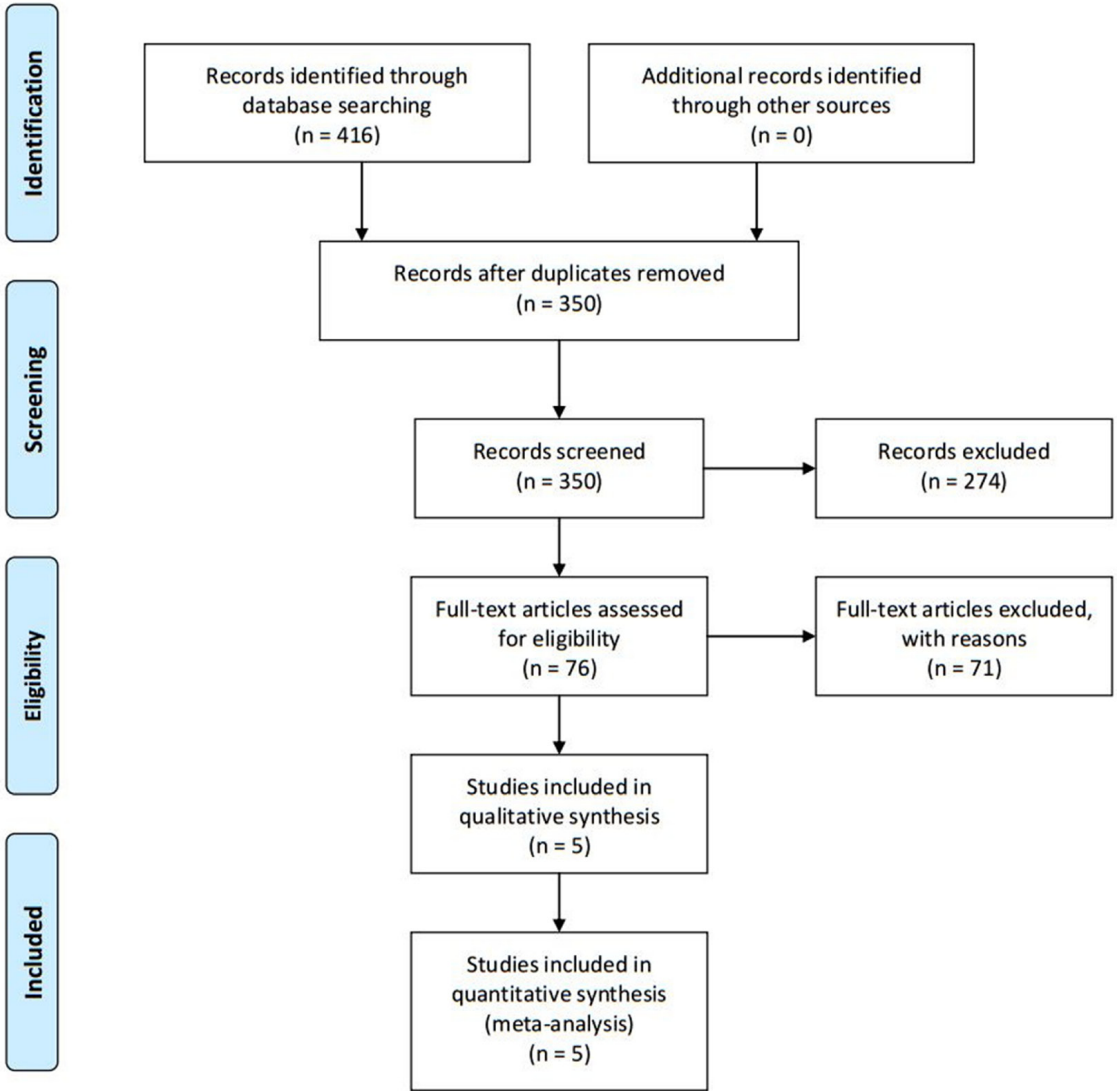
PRISMA flow diagram of literature search and selection

Figure 1 showed PRISMA flow diagram of studies identified by the search. The electronic database initially yielded 416 papers (151 papers in English and 265 in Chinese). Of these, 66 were subsequently removed due to duplication. The search identified 350 abstracts of which 76 were potentially relevant after title and abstract screening. Finally, five articles were included in our meta-analysis.

Studies were conducted in mainland China (Guangdong [6], Guangxi [11], Zhejiang [12], Jiangsu [10], and Inner Mongolia [13]). Among the 5 eligible studies published from 2011 to 2014. The sample sizes of the included studies ranged from 6304 to 158649 fetuses. These studies across 5 provinces/municipalities included a total of 393496 fetuses. The characteristics included in this meta-analysis were presented in Table 1. The diagnosis of CCAM was confirmed by high resolution ultrasound during pregnancy in all of the studies. Meanwhile, clinical-autopsy was also used to verify in fetus corpses in the five studies. However, only one study was reported that the pre-diagnostic CCAM newborns were demonstrated by computed tomography. Quality assessment scores of the included observational studies were also listed in Table 1. Four received 9 points and one received 8 points.

**Table 1:** Characteristics of the included studies on the prevalence of CCAM in China

Study	Year of Data Collection	*Maternal age (Years)	*Gestational Week	Province;Area	Cases	Sample size	Diagnostic method	NOS
Guo et al. 2014 [6]	200706–201206	…	…	Guangdong (S)	51	152166	US+CA	9
Yin et al. 2014 [10]	200806–201106	26.00 ± 0.50	25.00 ± 0.30	Jiangsu (Ce)	38	23617	US+CA+CT	9
Lin et al. 2011 [11]	200601–201008	17.00–42.00	9.00–43.00	Guangxi (S)	37	158649	US+CA	8
Wu et al. 2011 [12]	200701–201006	30.30 (18.00–45.00)	26.20 ± 7.00	Zhejiang (Ce)	6	52760	US+CA	9
Wang et al. 2011 [13]	200903–201003	32.50 (22.00–43.00)	11.00–40.00	Inner Mongolia (N)	5	6304	US+CA	9

CA, clinical-autopsy; CCAM, congenital cystic adenomatoid malformation; Ce, central; CT, Computed Tomography.

N, north; S, south; NOS, Newcastle-Ottawa Score; US, high-resolution ultrasound.

*Shown in mean ± standard deviation or minimum – maximum.

### Meta-analysis and sub-analysis

**Figure 2:**
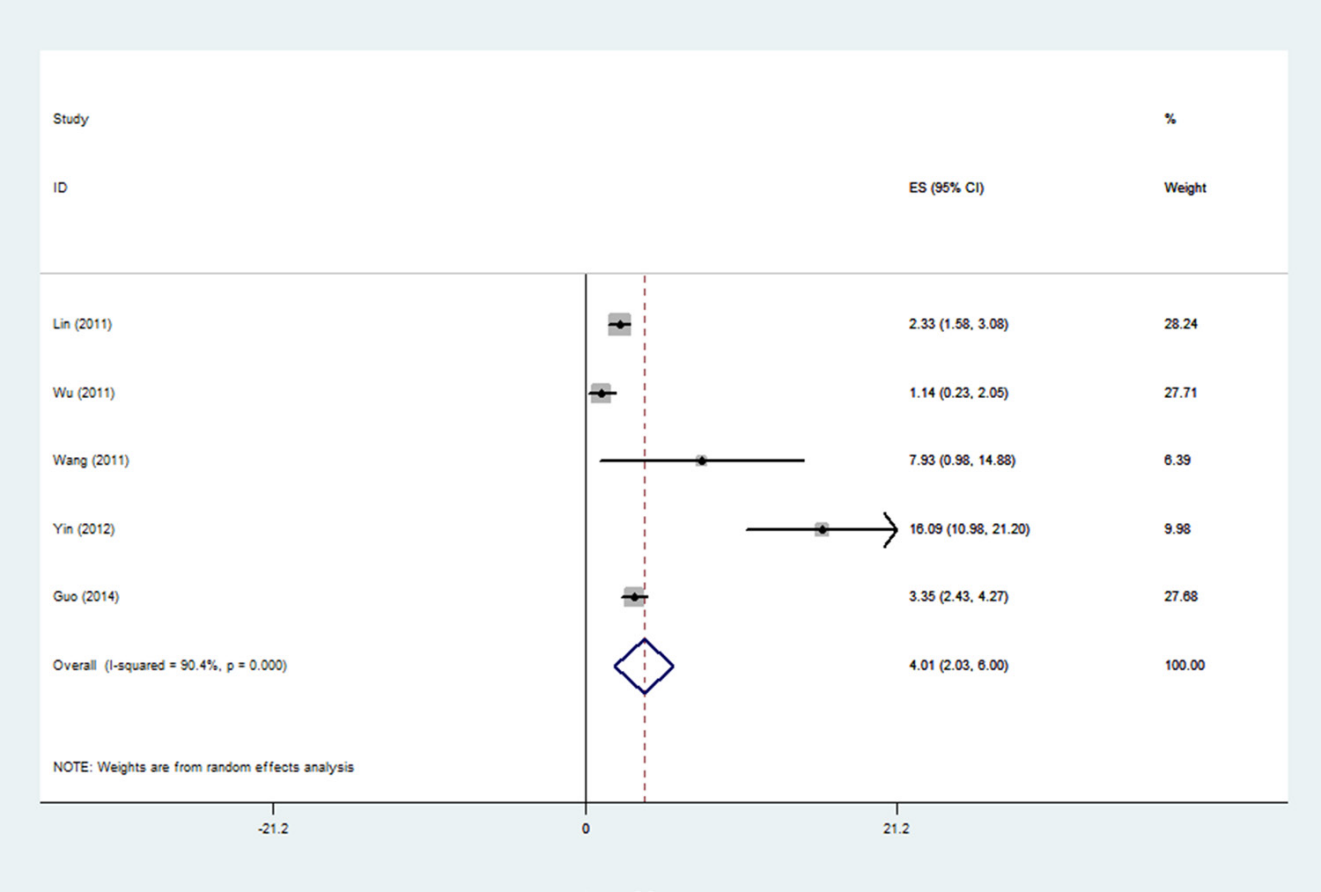
Forest plot of pooled estimated prevalence of CCAM in China with corresponding 95% confidence intervals

Table 2 summarized the overall and stratified pooled prevalence of CCAM by study characteristics such as study location, study region, sample size, and study quality. As showed in Table 2, the overall pooled prevalence of CCAM was 4.01/10000 (2.03/10000–6.00/10000) fetuses, characterized by high heterogeneity (*I*^2^ = 90.4, *p* < 0.0001) (Figure 2).

**Table 2:** Prevalence of CCAM among fetuses in China and subgroup analysis

Variable		Number of Surveys	Cases	Sample size	*P* (per 10000) [95%CI]	I^2^ (%)
Overall prevalence		5	137	393496	4.01 [2.03, 6.00]	90.4
Region	North	1	5	6304	7.93 [0.98, 14.88]	…
	Central	2	44	76377	8.93 [0.01, 23.04]	96.9
	South	2	88	310815	2.81 [1.81, 3.80]	90.4
						
Number	< 10000	1	5	6304	7.93 [0.98, 14.88]	…
	10000–100000	2	44	76377	8.39 [0.01, 23.04]	96.9
	≥ 100000	2	88	310815	2.81 [1.81, 3.80]	64.7
						
Quality Score	8	1	37	158649	2.23 [1.58, 3.08]	…
	9	4	100	234847	5.59 [2.35, 8.82]	92.8

Subgroup analyses based on geographical area showed that the CCAM prevalence was high in north (7.93/10000) and central (8.93/10000) areas, and low in south areas (2.81/10000). In the three number groups, the prevalence was lower (2.81/10000) for the studies with sample size over 100000, and higher in those with 10000–100000 (8.39/10000) and less than 10000 (7.93/10000) individuals. Higher quality score had a higher prevalence than the lower quality score (5.59/10000 vs. 2.23/10000). All of the subgroups were tested significant heterogeneity (Table 2).

Two studies [6, 10] reported the location of CCAM in fetal lung. The lesion was found easily occurred in the left lung. The ratio was 4:1 and 1.5:1, respectively. In the fetal sex, only one study [6] reported the information. The frequency was almost equally in the male (25) and female (26). Pregnancy outcomes were reported in two studies [6, 10]. In Guo et al study [6], twenty-four pregnant women were voluntary termination of pregnancy for misgiving the physical or mental health of her children; sixteen newborns were diagnosed with neonate pneumonia in the remaining twenty-seven newborns. In another study [10], twenty-nine pregnant women were voluntary termination of pregnancy, and three had a tendency for spontaneous decrease in size in the remaining nine newborns.

### Sensitivity analysis and publication bias

To confirm the stability and liability of the meta-analysis, sensitivity analysis was performed by repeating the calculation of pooled prevalence when any single study was deleted. Table 3 showed that the corresponding pooled prevalence ranged from 2.43/10000 (1.21/10000–3.67/10000) to 5.59/10000 (2.35/10000–8.23/10000).

**Table 3:** The results of the included studies through sensitivity analysis

Excluded study	Cases	Sample size	P (per 10000) (95%CI)
Before excluding	137	393496	4.01 (2.03–6.00)
Guo et al. 2014 [6]	86	241330	4.86 (2.02–7.70)
Yin et al. 2012 [10]	99	369879	2.43 (1.21–3.67)
Lin et al. 2011 [11]	100	234847	5.59 (2.35–8.23)
Wu et al. 2011 [12]	131	340736	5.46 (2.82–8.12)
Wang et al. 2011 [13]	132	387192	3.72 (1.70–5.75)

**Figure 3:**
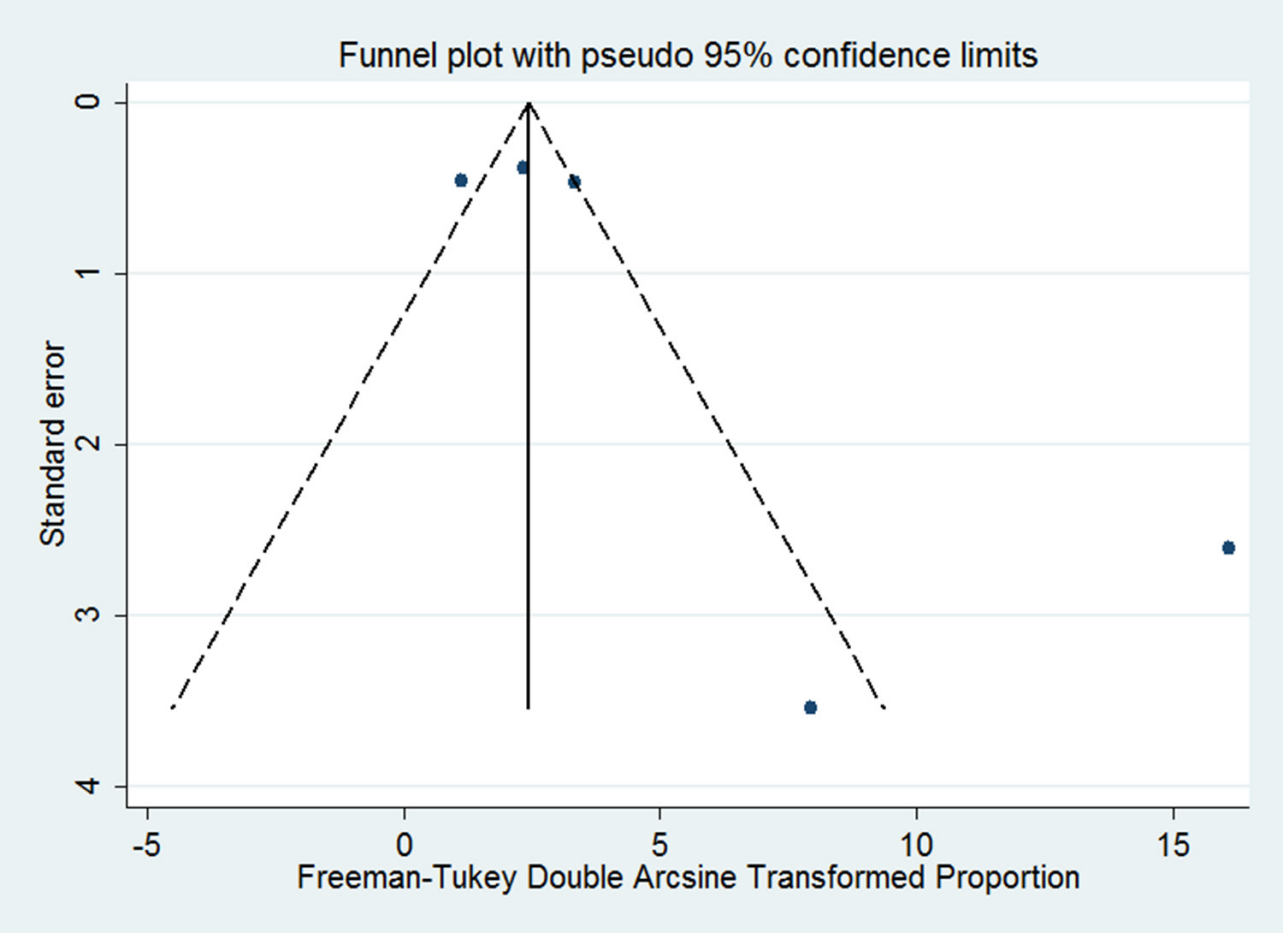
Funnel plot of the studies included in the meta-analysis

Funnel plots and the method of Begg’s and Egger’s test were performed to assess the publication bias of the study. Although the funnel plot was slightly asymmetrical (Figure 3), both Begg’s test (z = 0.73, *p* = 0.462) and Egg’s test (t = 1.86, *p* = 0.160) showed no potential risk of publication bias.

## DISCUSSION

To our knowledge, the analysis provided the first comprehensive, current estimated of the China prevalence of CCAM among fetuses as well its major epidemiological characteristics. In the current meta-analysis, a total of 393496 fetuses were included. It indicated that, among fetuses in China, the prevalence of CCAM ranged from 1.14/10000 to 16.09/10000, and the pooled prevalence was 4.01/10000.

It was reported that the prevalence of CCAM in developed country, such as Canada, was 1/25000 to 1/35000 among fetuses [5]. Our result was more than ten times higher than that in Canada. China is a vast country, and then the prevalence of CCAM is of significantly geographical distribution. During 2006–2012, Guangxi and Guangdong in southern China were provinces with lower prevalence of CCAM 2.33/10000, and 3.35/10000, respectively [6, 11]. However, Inner Mongolia (7.93/10000) and Jiangsu (16.09/10000) were higher prevalence provinces of CCAM [10, 13].

In this study, we also found that the prevalence of CCAM in north areas (7.93/10000) was higher than south areas (2.81/10000). It is very important to identify the geographical environmental risk factors of CCAM in the study area. The regional differences were attributed to a variety of reasons, such as economic status, health service status, diagnose level, ethnicity, and environmental pollution. As the largest developing country, China has achieved rapid economic development and urbanization process in east or coastal area; on the other hand, most of the western regions are still relatively poor.

More economically developed areas have better access to health care facilities. In this system review, only five studies reported the prevalence of CCAM among fetuses. All of them are from the eastern developed areas. The lack of balance and representatively made it extremely difficult to tell if the regional differences in prevalence were true or just a results of sampling bias. Therefore, it should be cautious and had better consider the confidence interval rather than the pooled result in the geographical distribution results.

Moreover, all of the five studies included in this meta-analysis were retrospective observational studies from out-patient data. None of them reported the potential factors which may influence the result except to the main information. Any influence of the above mentioned factors, including economic status, diagnose level, ethnicity, on regional variation of CCAM incidence is just speculative and not supported by the presented data. Then, a larger-scale multicenter, epidemiological study is needed for more persuasive analysis in the CCAM among fetuses in China.

There was only one study with sample size more than 100000 in this meta-analysis. Therefore, one potential reason for those heterogeneous findings may be that small sample sizes are more likely to lead to instable results, especially in the prevalence. Systematic review and meta-analysis provide a scientifically logical way to synthesize epidemiological data. Meta-analysis is a systematic approach to identifying, appraising, and synthesizing the results of relevant studies to make conclusions about a body of research, which could enhance the statistical power and draw a more reliable conclusion in comparison to a single study [14]. However, the comparison between the results of our meta-analysis of cross-sectional studies may not be correct because the prevalence of CCAM in the different regions of each province varies.

The study was strictly followed the MOOSE guidelines for reporting systematic reviews and meta-analysis, strict application of inclusion and exclusion criteria, using a critical appraisal of study quality, and up-to-date estimates using a DerSimonian-Laird random-effects model. Nevertheless, there were limitations due to the heterogeneity, and it should be considered when interpreting the findings of this study.

First, all of them were located in eastern China, while, data for western China was lacking, which meant that geographical distribution was unbalanced and the sample was not sufficiently representative. Therefore, it should be cautious and best to consider the confidence interval rather than the pooled result in the geographical distribution. Second, the diagnosis of CCAM may be dependent on the skill, experience and bias of the pathologist. Although all of the included studies were diagnosis using high resolution ultrasound during pregnancy and clinical-autopsy in fetus corpses, and each study may contain non-CCAM lung lesions that were diagnosed as CCAM. The results may be affected by the diagnosis bias. Third, heterogeneity was found in our meta-analysis of prevalence of CCAM, which may be attributed to the differences in sample size and areas. Last, because of the limited included studies, this study could not consider the interaction effect between different factors, such as maternal age, education, occupational exposure, ethnicity, lifestyle, economic level, and environmental pollution. Therefore, a large-scale multicenter study on the epidemiology of CCAM among fetuses across different areas in China is required.

In conclusion, our study pooled data from 393496 fetuses across five studies to provide the first estimates that reflect the present China burden and epidemiological characteristics of CCAM among fetuses. According to the results of this meta-analysis, the prevalence of CCAM among fetuses should be considerable in China.

## MATERIALS AND METHODS

The systematic review protocol has been published in the PROSPERO International Prospective Register of systematic reviews (http://www.crd.york.ac.uk/PROSPERO/), and the registration number is CRD42016045413. The present research performed a systematic review, in accordance to the Meta-analysis of observational studies in epidemiology (MOOSE) guidelines for systematic reviews of observational studies [15], and the Preferred Reporting Items for Systematic reviews and Meta-Analysis (PRISMA) statement for reporting systematic reviews and meta-analysis [16].

### Literature searches and selection

A systematic literature search was performed in four English (Pubmed, Elsevier Science Direct, Web of Science and Cochrane Library) and four Chinese (CBM, CNIK, VIP and Wanfang) databases for studies containing the data on the prevalence of CCAM among Chinese fetuses population, using a combined search strategy that included the following search terms: “congenital cystic adenomatoid malformation”, “CCAM”, “congenital pulmonary adenomatoid malformation”, “CPAM”, “prevalence”, “incidence”, “epidemiology”, “survey”, “China”, and “Chinese”. The databases were searched from their inception to July 2016 and the language restriction was not applied. The reference lists of retrieved articles were manually screened, and the results were also available to obtain the additional data by contacting original authors.

Original peer-reviewed publications were selected by two authors if they included: 1) > 1000 Chinese fetuses; 2) reported CCAM fetuses or newborns; 3) CCAM was diagnosed using high resolution ultrasound during pregnancy and confirmed after delivery using clinical-autopsy or Computed Tomography (CT). Studies were excluded based on the following criteria: 1) small sample size (less than 1000 fetuses), letters, reviews and editorials; 2) the full data was not accessible even after request from the corresponding/primary author. If multiple publications covered the same study, the most comprehensive one reporting the largest sample size was considered. This is a meta-analysis and approval by ethics committee or written consent could not require the extraction of data on the including studies.

### Data extraction

Two of the reviewers independently extracted the following information from each included study: the first author’s name, publication year, year of data collection, maternal age, gestational weeks, province or municipality name, study region (north, central, and south) [17], CCAM location (left or right lung), fetal sex, sample size, number of CCAM, diagnostic criteria, and pregnancy outcomes. A third reviewer confirmed all the extracted data. Missing raw data were requested from original authors by email from the corresponding/primary author.

### Quality assessment

Two review authors independently evaluated the methodological quality of the selected studies using the Newcastle-Ottawa Score (NOS) which was used to formally assess the quality of non-randomised cohort studies [18]. Each study was given a score of 0–9, based on the quality criteria.

### Statistical methods

The prevalence of CCAM in the selected studies was combined and reported as proportions with 95% CI using the STATA 12.0 (Stata-Corp, College Station, TX, USA). Before performing an inverse-variance weighted, the prevalence was transformed via the Freeman-Tukey double arcsine method [19]. The inverse variance methods and DerSimonian-Laird random-effects model meta-analysis were used to determine the weight of each study. Statistical heterogeneity was evaluated by the chi-square test of Q statistic, which was quantified by the I-square values, assuming that I-square values 25, 50 and 75% were nominally assigned as low, moderate, and high estimates, respectively [20]. To investigate potential sources of heterogeneity, subgroup analyses based on maternal age (< 25, 25–35 and > 35 years), study region (north, central and south), sample size (< 10000, 10000–100000 and >100000), study quality, and year of data collection (before 2000, 2001–2010, and after 2011) and meta-regression based on some of methodological factors and study population characteristics were performed to assess the association between these variables and the prevalence estimates. Furthermore, sensitivity analysis was also performed to examine the influence of excluding some specific studies on the overall estimates. Finally, a funnel plot (prevalence versus standard error) and the method of Begg’s and Egger’s test were used to explore the potential publication bias. *P* ≤ 0.05 indicated that it was statistically significant.

## References

[1] Hellmund A, Berg C, Geipel A, Bludau M, Heydweiller A, Bachour H, Muller A, Muller A, Gembruch U (2016). Prenatal Diagnosis and Evaluation of Sonographic Predictors for Intervention and Adverse Outcome in Congenital Pulmonary Airway Malformation. PloS one.

[2] Ch’In KY, Tang MY (1949). Congenital adenomatoid malformation of one lobe of a lung with general anasarca. Arch Pathol (Chic).

[3] Stocker JT, Madewell JE, Drake RM (1977). Congenital cystic adenomatoid malformation of the lung. Classification and morphologic spectrum. Hum Pathol.

[4] Stocker JT (2002). Congenital pulmonary airway malformation: a new name and an expanded classification of congenital cystic adenomatoid malformation of the lung. Histopathology.

[5] Laberge JM, Flageole H, Pugash D, Khalife S, Blair G, Filiatrault D, Russo P, Lees G, Wilson RD (2001). Outcome of the prenatally diagnosed congenital cystic adenomatoid lung malformation: a Canadian experience. Fetal Diagn Ther.

[6] Guo XL, Wang Q, Liu ZP, Suo DM, Zeng M, Huang Y, Feng JP (2012). Clinical analysis on prevalence and pregnancy outcome of fetal congenital cystic adenomatoid malformation in Foshan from 2007 to 2012. MCHCC.

[7] Xie D, Yang T, Liu Z, Wang H (2016). Epidemiology of Birth Defects Based on a Birth Defect Surveillance System from 2005 to 2014 in Hunan Province, China. PloS one.

[8] Yang M, Zhang S, Du Y (2015). Epidemiology characteristics of birth defects in Shenzhen city during 2003 to 2009, China. J Matern Fetal Neonatal Med.

[9] Dai L, Zhu J, Liang J, Wang YP, Wang H, Mao M (2011). Birth defects surveillance in China. World J Pediatr.

[10] Yin LL, Deng XD, Tang YQ, Ling C, Liang H, Jiang XL (2012). Prenatal diagnosis and typing of fetal cystic adenomatoid malformation of the lung by ultrasound. Chin J Med Ultrasound.

[11] Lin LE, Tian XX, Liang JM (2011). Ultrasonic diagnosis of 1670 fetuses with fetal malformation. MCHCC.

[12] Wu FX, Peng XH, Huang WF, Hong LX (2011). Analysis of prenatal ultrasound findings of fetal abnormality from 2007 to 2010 in Taizhou hospital. MCHCC.

[13] Wang L, Luo L, Shao ZY, He XH, Xu ZH, Ma XH, Shang Y (2011). Clinical analysis of 6034 fetus by prenatal ultrasound screening. Inner Mongolia Med J.

[14] Fan D, Liu L, Ding N, Liu S, Hu Y, Cai G, Xia G, Xin L, Wang L, Xu S, Xu J, Zou Y, Pan F (2015). Male sexual dysfunction and ankylosing spondylitis: a systematic review and metaanalysis. J Rheumatol.

[15] Stroup DF, Berlin JA, Morton SC, Olkin I, Williamson GD, Rennie D, Moher D, Becker BJ, Sipe TA, Thacker SB (2000). Meta-analysis of observational studies in epidemiology: a proposal for reporting. Meta-analysis Of Observational Studies in Epidemiology (MOOSE) group. Jama.

[16] Moher D, Liberati A, Tetzlaff J, Altman DG, PRISMA Group (2010). Preferred reporting items for systematic reviews and meta-analyses: the PRISMA statement. Int J Surg.

[17] Yang B, Fan S, Zhi X, Wang Y, Wang Y, Zheng Q, Sun G (2015). Prevalence of hyperhomocysteinemia in China: a systematic review and meta-analysis. Nutrients.

[18] Kyrgiou M, Athanasiou A, Paraskevaidi M, Mitra A, Kalliala I, Martin-Hirsch P, Arbyn M, Bennett P, Paraskevaidis E (2016). Adverse obstetric outcomes after local treatment for cervical preinvasive and early invasive disease according to cone depth: systematic review and meta-analysis. Bmj.

[19] Freeman MF, Tukey JW (1950). Transformations Related to the Angular and the Square Root. Ann Math Statist.

[20] Fan DZ, Wu S, Wang W, Xin LH, Tian G, Liu L, Feng JP, Guo XL, Liu ZP (2016). Prevalence of placenta previa among deliveries in Mainland China: A PRISMA-compliant systematic review and meta-analysis. Medicine.

